# Pixel level anomaly detection in second harmonic generation microscopy for non destructive defect inspection of AlGaN/GaN heterostructures

**DOI:** 10.1186/s11671-026-04717-0

**Published:** 2026-06-11

**Authors:** Chen-Fang Kang, Chih-Chang Hung, Che-Hsuan Huang, Ting-Chuan Li, Wu-Yih Uen, Ching-Hsueh Chiu

**Affiliations:** 1 Department of Electronic Engineering, Chung Yuan Christian University, Taoyuan, 320314 Taiwan; 2 Research Center for Semiconductor Materials and Advanced Optics, Chung Yuan Christian University, Taoyuan, 320314 Taiwan; 3 Bolite Co., Ltd, Zhubei, Hsinchu County 305042 Taiwan; 4 LEDA Creative technology LTD, Taoyuan, 320016 Taiwan

**Keywords:** Second harmonic generation, Cathodoluminescence, Defects, Pixel level anomaly detection, GaN, Nonlinear optics

## Abstract

In this study, we present a semi-supervised inspection framework that integrates second harmonic generation (SHG) microscopy with pixel-level anomaly detection (PLAD) for high-sensitivity defect mapping in AlGaN/GaN heterostructures grown on Si (111). SHG is intrinsically sensitive to local inversion symmetry breaking and modulation of the effective second-order nonlinear susceptibility. As a result, spatial variations in the nonlinear optical response serve as a physical contrast mechanism for detecting structural inhomogeneities. The SHG-derived defect features are further supported by correlation with cathodoluminescence (CL) based R_DE/NBE_ defect metrics. Complementary to conventional supervised object-centric detectors such as YOLOv8 and Mask R-CNN, the PLAD approach statistically models nominal lattice patterns without manual annotations. This strategy enables improved sensitivity to weak and spatially diffuse crystalline irregularities. Quantitative evaluation demonstrates substantially improved spatial overlap and detection sensitivity compared with object-based models, resolving more than 5,000 micron-scale defect-affected regions within a single field of view that are largely inaccessible to bounding-box or mask-based architectures. By bridging nonlinear optical microscopy with pixel-level anomaly modeling, the SHG-PLAD framework provides a non-contact strategy for defect-sensitive inspection and offers a useful approach for crystalline defect characterization and quality assessment in AlGaN/GaN heterostructures.

## Introduction

III-nitride semiconductors, particularly GaN and its alloys, have attracted substantial interest due to their wide bandgap, high breakdown field, and exceptional thermal stability, positioning them as premier materials for high-power and high-frequency applications. In AlGaN/GaN heterostructures, spontaneous and piezoelectric polarization effects induce a significant built-in electric field, leading to the formation of a triangular quantum well at the heterointerface. This confinement generates a high-density two-dimensional electron gas (2DEG) with superior carrier mobility, which is fundamental to the performance of high electron mobility transistors (HEMTs) [1–3]. However, each type of defect negatively impacts wafer quality, leading to the degradation of devices fabricated on it. The correlation between defects and device failures is quantified by the kill ratio, defined as the proportion of defects estimated to induce device failure [4]. Thus, those defects that cause significant impact on the device are referred to as killer defects [5]. Following epitaxial layer growth, the wafer undergoes reinspection to precisely locate defects and verify that defect densities remain within acceptable limits.

Recent advancements in lattice defect characterization for epi-wafer have underscored the indispensable role of high-resolution X-ray diffraction (HR-XRD), X-ray topography, and atomic resolution transmission electron microscopy (TEM) in revealing dislocation structures, strain distributions, and nanoscale defect dynamics. However, these techniques require a high vacuum environment and exhibit relatively slow imaging speeds, making rapid and large area defect characterization a persistent challenge. To accurately identify both the type and spatial distribution of crystalline defects in epitaxial wafers, potassium hydroxide (KOH) is often employed as the etchant for defect revelation etching. As a result, the etched patterns of typical defects such as threading dislocations (TDs), stacking faults, and grain boundaries can be easily distinguished using conventional optical microscopy [6–7]. While KOH etching enables rapid defect mapping, it is inherently destructive and alters the surface morphology of the wafer. As a result, epi-wafers subjected to KOH treatment are no longer suitable for subsequent device fabrication or electrical characterization. This would limit the applicability of KOH etching in in-line quality control and process monitoring. Meanwhile, optical measurement techniques, being fast, non-contact, and nondestructive, are highly suitable for in-situ and large-area inspection in industrial production lines. They can be seamlessly integrated into manufacturing workflows to enable real time monitoring of defect distributions and crystalline uniformity without interrupting the fabrication process [8–10]. Single photon excited (SPL) and two-photon excited photoluminescence (2PPL) are two typical measurements used to probe defect states in semiconductor epilayers [11–13]. However, prior to radiative recombination via band-edge, phonon-assisted, or defect-assisted transitions, photoexcited carriers in crystalline materials must undergo an incoherent thermalization process [14]. This carrier relaxation introduces significant complexity in interpreting the resultant emission dynamics, thereby limiting the precision of defect attribution [15]. Second harmonic generation (SHG), a specific case of sum-frequency generation, is one of the most prominent and widely utilized nonlinear optical (NLO) effects. In practical applications, non-centrosymmetric semiconductors exhibiting strong SHG responses play a critical role by the effective second order nonlinear susceptibility χ^(2)^. They are essential in the development of advanced optical and electro-optical devices, including frequency-doubled lasers, modulators, and nonlinear wavelength converters [16]. Importantly, the presence of structural defects locally breaks the inversion symmetry of the crystal lattice. This alteration affects the effective second order nonlinear susceptibility and leads to spatial variations in SHG intensity. This sensitivity makes SHG a valuable non-invasive tool for probing defect-induced symmetry breaking in crystalline materials [17]. Due to its non-centrosymmetric wurtzite crystal structure, GaN exhibits strong second order nonlinear optical properties. This structure enables efficient SHG through direct photon crystal interactions [18–20].

Despite the growing adoption of machine learning for automated defect detection, accurately identifying microscopic crystalline irregularities remains challenging. Conventional supervised object-centric architectures, such as YOLOv8 and Mask R-CNN, are primarily optimized for macroscopic features with well-defined boundaries and therefore often struggle to detect subtle, spatially diffuse anomalies typical of compound semiconductor systems. In III-nitride heterostructures, defect signatures frequently manifest as weak contrast variations embedded within complex nonlinear optical signals, making them particularly susceptible to background noise and ambiguity in object-based representations. Recent advances in semi-supervised anomaly detection provide an alternative pathway by modeling the statistical characteristics of nominal material responses without requiring extensive manual annotation. In this work, we introduce a physics-informed inspection framework that integrates SHG microscopy with pixel-level anomaly detection (PLAD) to achieve high-sensitivity defect mapping in AlGaN/GaN heterostructures, combining physical interpretability with data-driven adaptability and enabling early-stage defect characterization prior to device fabrication.

## Experimental

### Second harmonic generation

Second harmonic generation (SHG) is a two-photon, frequency-doubling nonlinear optical process that occurs exclusively in non-centrosymmetric materials. Under the electric-dipole approximation, the intensity of the generated second-harmonic (2ω) signal can be expressed as Eq. (1):

1 $$\:{\boldsymbol{I}}_{2\boldsymbol{\omega\:}}\propto\:{\left|{\boldsymbol{P}}^{\left(2\right)}\left(2\boldsymbol{\omega\:}\right)\right|}^{2}={\left|{\boldsymbol{ϵ}}_{0}\sum\:_{\boldsymbol{j},\boldsymbol{k}}{\boldsymbol{\upchi\:}}_{\boldsymbol{i}\boldsymbol{j}\boldsymbol{k}}^{\left(2\right)}{\boldsymbol{E}}_{\boldsymbol{j}}\left(\boldsymbol{\omega\:}\right){\boldsymbol{E}}_{\boldsymbol{k}}\left(\boldsymbol{\omega\:}\right)\right|}^{2}$$

where *I*_*2ω*_ and *P*^*(2)*^*(2ω)* denote the intensity and polarization vector of the second-harmonic light, respectively; $\:{\boldsymbol{ϵ}}_{0}$ the vacuum permittivity; and $\:{\chi\:}_{ijk}^{\left(2\right)}$enotes the elements of the second-order nonlinear susceptibility tensor (a third-rank tensor), which is intrinsically sensitive to the local symmetry of the crystal lattice; E_j_(ω) and E_k_(ω) are the electric field components of the fundamental incident wave [21]. Crucially, the effective$\:{\chi\:}^{\left(2\right)}$ tensor is a macroscopic manifestation of the long-range order in a non-centrosymmetric lattice. The presence of crystalline defects modifies the effective second-order nonlinear susceptibility $\:{\chi\:}^{\left(2\right)}$, leading to local variations in SHG intensity depending on defect type and strain distribution. Therefore, spatial variations in *I*_*2ω*_ provide a high contrast optical proxy for mapping the density of structural inhomogeneities within the AlGaN/GaN heterostructure.

### Sample preparation

**Fig. 1:**
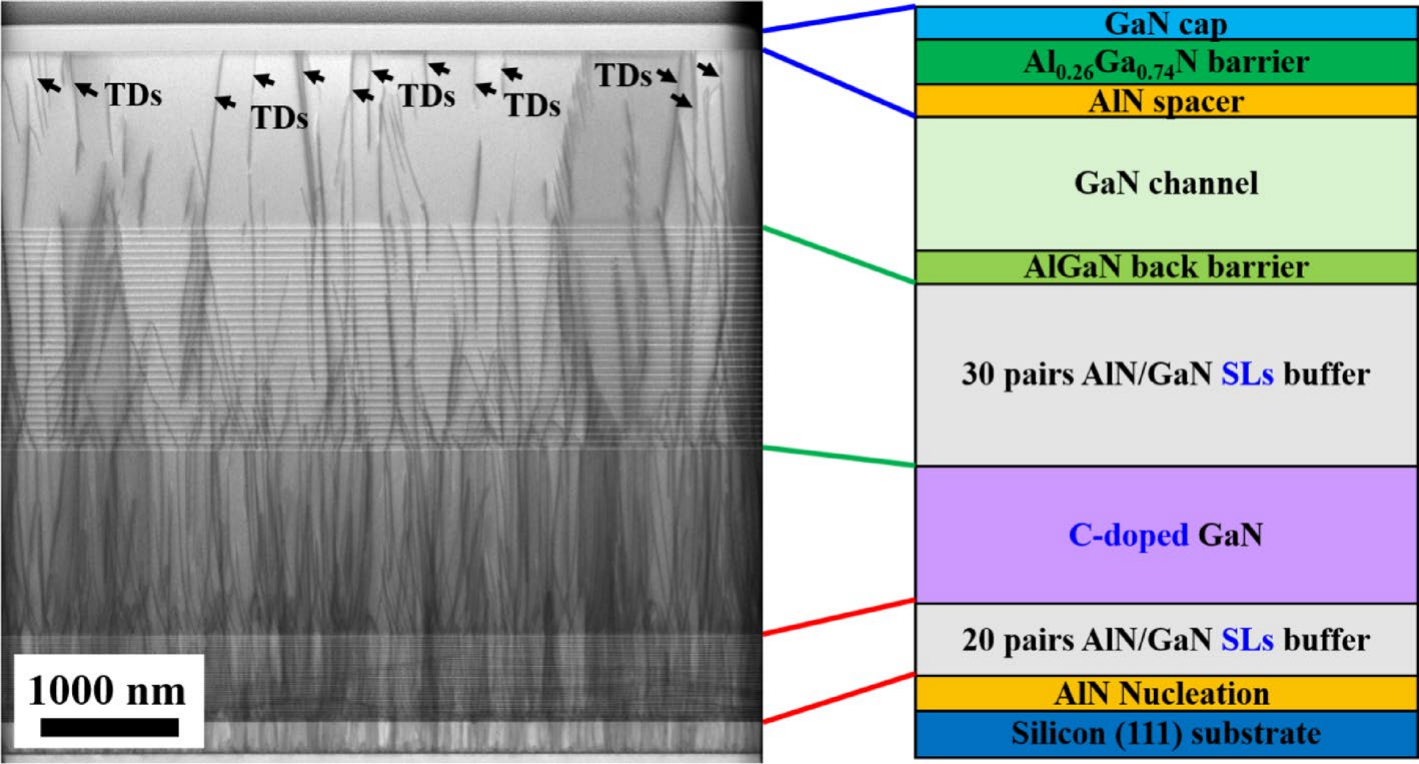
The cross-sectional TEM image of the AlGaN/GaN heterostructure accompanied by the schematic of sample to denote the corresponding layer details

The heterostructure analyzed in this work was grown by metal organic chemical vapor deposition (MOCVD) on Si (111) substrate. The layer structure was deposited sequentially by an AlN nucleation layer, 20 pairs AlN/GaN superlattices (SLs) buffer layer, 1.2 μm of C-doped GaN, 30 pairs AlN/GaN superlattices (SLs) buffer layer, 1.2 μm of undoped GaN (u-GaN), 1 nm AlN layer, 25 nm of Al_0.26_Ga_0.74_N layer, and topped by a 15-nm-thick u-GaN capping layer. The cross-sectional TEM image of the AlGaN/GaN epitaxial structure is shown in Fig. 1. In the same figure, a schematic diagram of the sample is accompanied to indicate the corresponding layer details. The cross-sectional TEM image confirms the successful growth of the AlGaN/GaN heterostructure. Notably, the TDs are visible as dark contrast lines originating from the AlN/GaN superlattices and propagating through the GaN channel layer. These structural imperfections locally disrupt the crystalline symmetry, serving as the physical basis for the symmetry breaking induced contrast observed in the subsequent SHG mapping analysis.

### The measurement system

**Fig. 2:**
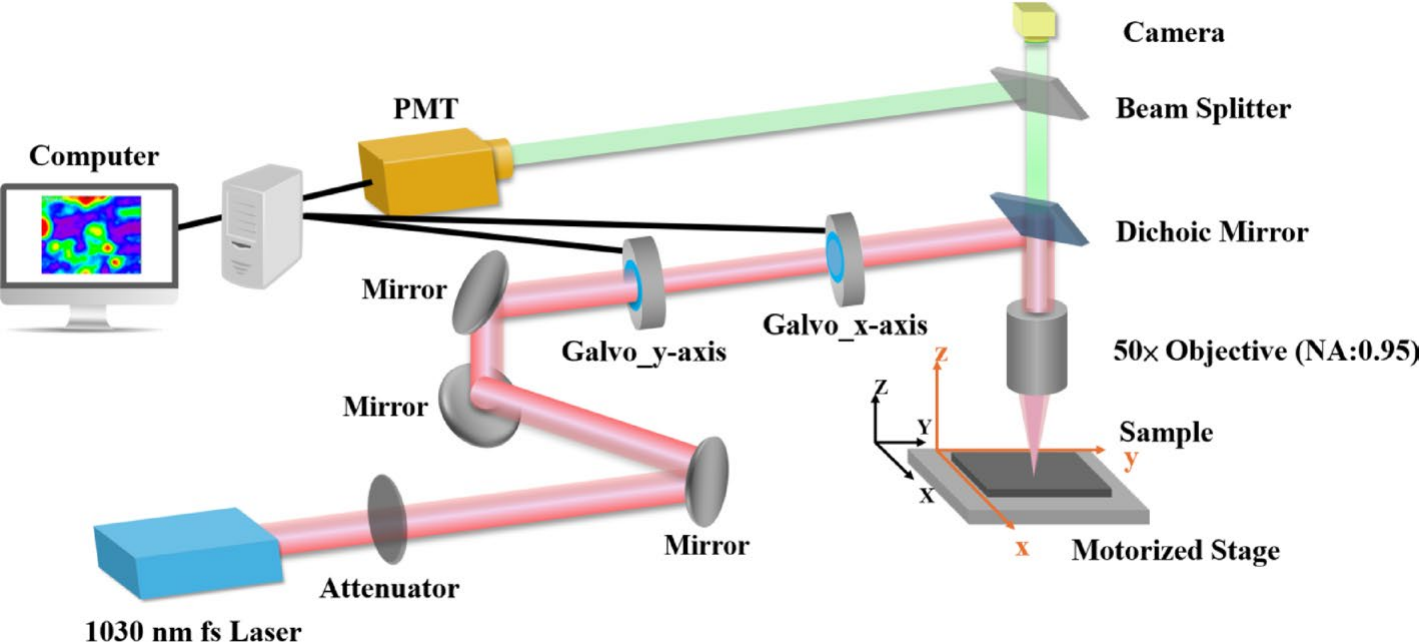
A schematic illustration of the home-designed SHG microscopy

Figure 2 illustrates the configuration of the second-harmonic generation (SHG) microscopy system employed in this study. The excitation source is a 1030 nm femtosecond pulsed laser (mRadian, Kasmoro), which is directed through a pair of galvanometric mirrors for beam steering. The beam is relayed by a lens system and tightly focused onto the sample surface using a 50× long-working-distance objective lens with a numerical aperture (NA) of 0.95. Under these conditions, diffraction-limited focusing results in a lateral spot size of approximately 1 μm at the focal plane, enabling raster scanning in the x-y plane with micrometer-scale spatial resolution. Z-axis positioning is achieved by vertically translating the sample using a high-precision motorized stage. Owing to the high-NA focusing geometry, the axial resolution is estimated to be on the order of a few micrometers (~ 2–3 μm), based on Gaussian beam optics. Accordingly, the detected SHG signal represents an effective second order nonlinear optical response integrated over the excitation focal volume rather than a strictly layer-specific contribution. Given that threading dislocations and their associated strain fields extend vertically across multiple epitaxial layers in AlGaN/GaN heterostructures, the measured SHG contrast reflects the cumulative modulation of the effective nonlinear susceptibility within this sampled volume. The generated 515 nm SHG signal is collected in an epi-detection configuration through the same objective lens. The collected light is routed through a dichroic mirror and short-pass filters to suppress residual 1030 nm excitation before being detected by a photomultiplier tube (PMT; Hamamatsu H7422) operating in photon-counting mode. To ensure crystallographic consistency, the samples were mounted with the [0001] c-axis aligned along the laboratory Z-axis during measurement.

For independent validation of the SHG contrast mechanism, spatially resolved cathodoluminescence (CL) imaging was performed using an Attolight Allalin system. The measurements were conducted at an accelerating voltage of 5 keV and a probe current of 30 nA. Under these conditions, the effective CL lateral resolution is well within the SHG sampling scale, enabling reliable spatial correlation between nonlinear optical response and defect-related luminescence. To achieve precise pixel-wise alignment between the SHG and CL datasets, a feature-based image registration strategy was implemented. Distinct morphological landmarks and localized defect clusters identified in both modalities were used as control points to determine the optimal spatial transformation parameters. To compensate for differences in magnification and orientation between the laser scanning SHG and electron beam-based CL systems, a linear affine transformation (including scaling, rotation, and translation) was applied. The registration accuracy was verified by minimizing the residual positional error between corresponding landmarks, thereby ensuring consistent spatial correspondence for subsequent correlation analysis.

### Architecture workflow

**Fig. 3:**
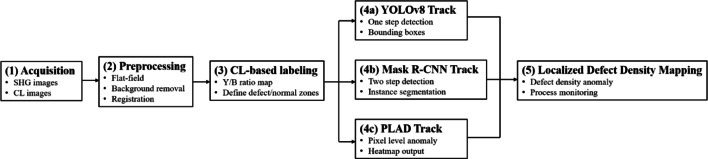
Workflow of the proposed SHG/CL-based defect inspection pipeline, consisting of: **1** acquisition of SHG and CL images; **2** preprocessing, including flat-field correction, background removal, and image registration;** 3** CL-based labeling for training data generation; **4a** YOLOv8 detection track, **4b** Mask R-CNN detection track, and **4c** PLAD anomaly detection track; and **5** localized defect density mapping for defect characterization and quality assessment

We propose an integrated inspection framework that synergizes multimodal optical imaging with advanced machine learning paradigms to achieve high-resolution, non-destructive characterization of AlGaN/GaN heterostructures. As illustrated in Fig. 3, the workflow is executed through a sequence of five specialized stages:

1. Multimodal Image Acquisition: High-resolution SHG and hyperspectral CL intensity mappings are collected from identical localized regions of interest.
2. Data Preprocessing and Co-registration: Raw datasets undergo flat-field correction and background subtraction. To ensure absolute spatial correspondence between optical and electron-beam-derived maps, a feature based affine registration strategy is implemented. This procedure determines optimal spatial transformation parameters, achieving precise pixel-wise alignment across different imaging modalities.
3. Physical Ground Truth (GT) Generation: Quantitative GT labels are derived from CL hyperspectral data by calculating the Yellow to Band edge (Y/B) emission ratio. This ratio serves as a robust physical proxy for localized variations in defect density, providing a scientific baseline for evaluating model performance.
4. Multi-track Defect Inspection: The architecture facilitates a comparative analysis across three distinct detection tracks: a single-stage YOLOv8 bounding-box detector, a two-stage Mask R-CNN instance segmentation model, and the proposed semi-supervised PLAD framework, which generates fine-grained anomaly score heatmaps without the requirement for manual annotations.
5. Localized Defect Density Mapping: Finally, the outputs from the algorithmic tracks are synthesized into localized defect density maps. By quantifying the spatial distribution of identified anomalies within the scanned micro-regions, this stage provides high-resolution feedback on structural inhomogeneities and crystalline quality.

### Pixel level anomaly detection

**Fig. 4:**
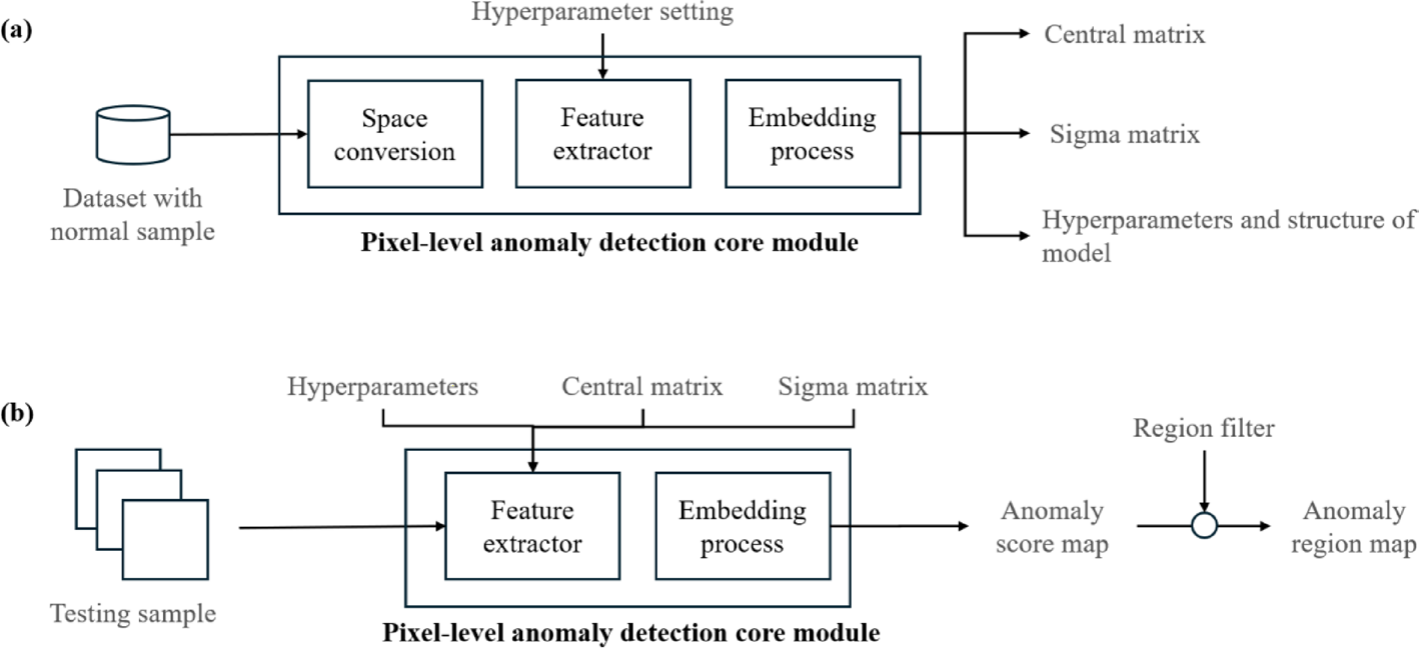
**a** Embedding process of the PLAD core module, showing space conversion, feature extraction, and embedding of normal samples. **b** Inference process of the PLAD module, where testing samples are processed to generate anomaly score maps and anomaly region maps

The PLAD framework is designed to achieve fine-grained localization of structural irregularities by modeling the statistical distribution of “normal” (defect-free) crystalline patterns. Unlike conventional supervised object detectors that require extensive manual labeling of bounding boxes, PLAD operates on a semi-supervised learning paradigm, making it highly effective for identifying previously unseen or irregularly shaped defects that deviate from nominal lattice symmetry. The architecture of the PLAD core module is illustrated in Fig. 4. The process is partitioned into two primary stages:

- Normality Modeling (Training Stage): In this study, the normality model was established using a dataset of 200 defect-free SHG image regions (160 × 160 μm²). These defect-free regions were selected based on low R_DE/NBE_ values confirmed by spatially resolved CL mapping, ensuring minimal defect-related luminescence contribution. The images were then augmented and subdivided into thousands of localized patches for multi-scale feature extraction. Utilizing a fixed Wide-ResNet50 convolutional neural network (CNN) backbone, the system extracts high-dimensional embeddings to construct a central matrix and a sigma matrix, which represent the statistical baseline of a high quality GaN lattice.
- Anomaly Inference (Testing Stage): During inference, embeddings from testing samples are projected into the learned feature space and evaluated against the modeled normal distribution to generate a pixel wise anomaly score map. To refine the detection results, post processing operations, including median filtering and connected component analysis, are applied to produce the final anomaly region map.

### The 8-connected component labeling (CCL) technique

To quantitatively assess the efficacy of defect region annotation and detection, an 8-connected component labeling (8-CCL) technique was utilized to extract spatially contiguous regions from the binarized or color-masked model outputs [22]. Within a connectivity framework, pixels are deemed connected if they share either an edge or a vertex. Formally, two pixels, denoted as *p* at ($\:{\boldsymbol{x}}_{\boldsymbol{i}}$, $\:{\boldsymbol{y}}_{\boldsymbol{i}}$) and *q* at ($\:{\boldsymbol{x}}_{\boldsymbol{j}}$, $\:{\boldsymbol{y}}_{\boldsymbol{j}}$) are considered part of the same contiguous region if they satisfy the following condition:

2 $$ ~\max \left( {\left| {x_{i} - x_{j} } \right|,~\left| {y_{i} - y_{j} } \right|} \right) \le $$

For each color or detection channel, a binarized mask ***M(x***,*** y)*** is defined as:

3 $$ ~M\left( {x,y} \right) = \left\{ {\begin{array}{*{20}c} {1,~~if~the~pixel~belongs~to~annotation~region~~~~~~~~} \\ {0,~~otherwise~~~~~~~~~~~~~~~~~~~~~~~~~~~~~~~~~~~~~~~~~~~~~~~~~~~~~~~~~~~~~~~~~~} \\ \end{array} } \right. $$

- The total area of each connected region ***R***, denoted as ***A(R)***, is calculated by integrating the foreground pixels within that region, which can be expressed by Eq. (4):

4 $$ ~A\left( R \right) = \mathop \sum \limits_{{\left( {x,y} \right) \in R}} M\left( {x,y} \right) $$

- where ***A(R)*** represents the total count of pixels constituting region ***R***. This methodology facilitates a uniform, pixel-level morphological comparison across the YOLOv8, Mask R-CNN, and PLAD models.
- To further quantify the agreement between the predicted defect regions and the CL-derived reference map, several pixel-level evaluation metrics were employed in this study. The area ratio was used to compare the total detected defect area with the reference defect area, and is defined as:

5 $$ Area~Ratio = ~\frac{{A_{{pred}} }}{{A_{{GT}} }} $$

- where *A*_*pred*_ is the total number of pixels classified as defect by the model, and *A*_*GT*_ is the total number of pixels labeled as defect in the CL-derived reference map. An area ratio greater than 1 indicates overestimation of defect regions, whereas a value smaller than 1 indicates underestimation.

The pixel-wise accuracy is defined as the proportion of correctly classified pixels (both defect and non-defect) over the total number of pixels, which can be expressed by Eq. (6):

6 $$\:\:Pixel-wise\:accuracy=\:\frac{TP+TN}{TP+TN+FP+FN}$$

- where TP, TN, FP, and FN denote the numbers of true positive, true negative, false positive, and false negative pixels, respectively.

Intersection over Union (IoU) evaluates the spatial overlap between the predicted defect regions and the ground truth. It is defined as:

7 $$\:IoU=\:\frac{TP}{TP+FP+FN}$$

IoU measures how well the predicted defect regions align with the reference defect map, taking into account both overestimation (false positives) and underestimation (false negatives). A higher IoU indicates better spatial consistency and more accurate localization of defect regions.

Precision quantifies the reliability of the detected defect pixels by measuring the proportion of predicted defect pixels that are truly defective according to the ground truth. It is defined as:

8 $$\:Precision=\:\frac{TP}{TP+FP}$$

A higher precision indicates that the detected defect regions contain fewer false alarms, reflecting better specificity of the detection method.

Recall, also referred to as sensitivity, measures the ability of the model to identify actual defect pixels. It is defined as:

9 $$\:Recall=\:\frac{TP}{TP+FN}$$

A higher recall indicates that the method can capture a larger fraction of true defects, which is particularly important for detecting weak or spatially diffuse anomalies in inspection.The F1-score provides a balanced measure that considers both precision and recall. It is defined as the harmonic mean of precision and recall:

10 $$ F1 - score{\text{ }} = ~\frac{{2 \cdot \Pr ecision \cdot \mathrm{Re} call}}{{\Pr ecision + \mathrm{Re} call}} $$

The F1-score is especially useful when there is an imbalance between defect and non-defect pixels, as it penalizes extreme trade-offs between precision and recall. A higher F1-score indicates a more balanced and robust detection performance.

## Results and discussions

### CL-derived defect mapping

**Fig. 5:**
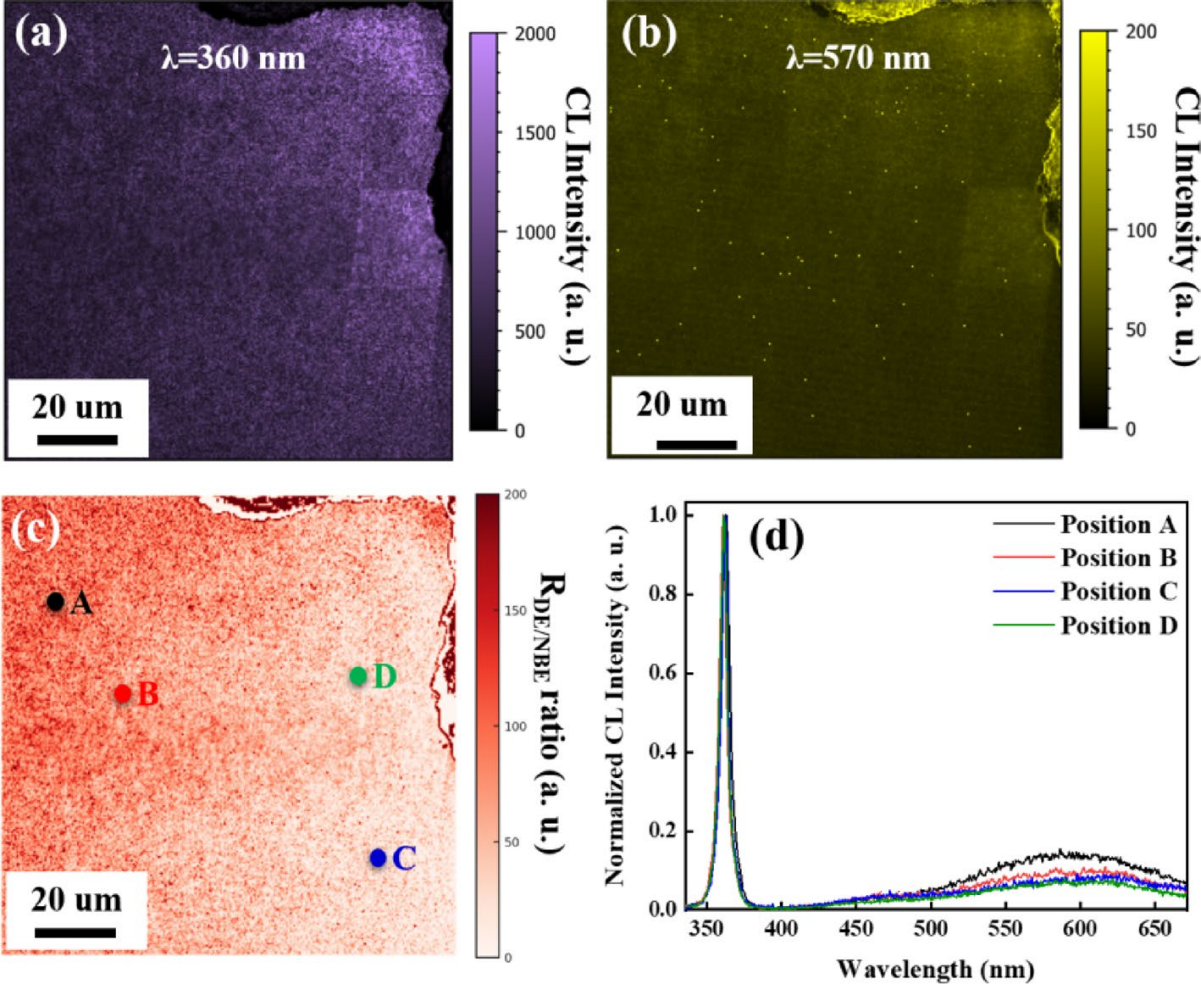
Monochromatic images extracted from the hyperspectral data set showing the CL intensity mapping at **a** 360 nm and **b** 570 nm for NBE and defect-related emissions, respectively; **c** the CL intensity mapping of DE/NBE ratio; **d** CL spectra of AlGaN/GaN heterostructure taken at various positions

Figure 5 provides the CL spectroscopic foundation for defect identification within the GaN-on-Si epilayers. A sharp near-band-edge (NBE) emission centered at 360 nm is observed, consistent with the intrinsic band-to-band recombination of GaN reported previously [23]. In contrast, a broad deep-level emission (DE) band peaking near 570 nm is also found. The latter is commonly referred to as yellow luminescence (YL) and associated with Ga vacancy related complexes and other point defects [24]. Furthermore, monochromatic CL intensity mappings conducted at wavelengths of 360 nm and 570 nm are documented in Figs. 5(a) and 5(b), respectively. These mappings reveal pronounced spectral characteristics indicative of the intrinsic crystalline quality of the GaN epilayer. To quantitatively assess the spatial distribution of defect induced recombination, the ratio of CL intensity mappings is compared using Eq. (11):

11 $$ ~R_{{DE/NBE}} \left( {x,y} \right) = \frac{{I_{{DE}} \left( {x,y} \right)}}{{I_{{NBE}} \left( {x,y} \right)}} $$

Where I_DE_ is the CL intensity around 570 nm and I_NBE_ is that of the NBE emission near 360 nm. The resulting map of R_DE/NBE_ is depicted in Fig. 5(c). The regions highlighted with higher R_DE/NBE_ values would suggest increased nonradiative recombination centers and inferior local crystalline quality. The CL spectra taken at various locations on the sample denoted as A, B, C, and D are stacked together in Fig. 5(c). Sites A and B exhibit high R_DE/NBE_ values and dominant YL features, indicative of substantial defects-related emissions. In contrast, spectra from C and D demonstrate lower values of R_DE/NBE_ and prominent NBE emission, confirming superior material quality at these locations. These findings are in excellent agreement with the CL spectral profiles shown in Fig. 5(d), which clearly differentiate high-defect and low-defect regions through their respective emission characteristics. This CL-derived ratio mapping establishes the physical GT necessary for evaluating the subsequent nonlinear optical characterization.

### Correlation between defect-induced SHG modulation and CL-derived defect mapping

**Fig. 6:**
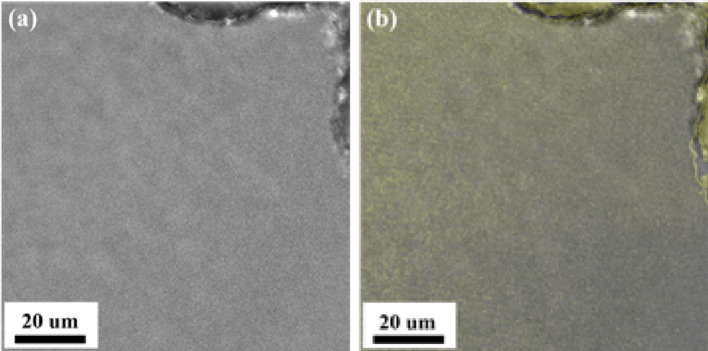
**a** SHG image of GaN-on-Si epilayer; **b** Overlay of the R_DE/NBE_ value of CL map onto corresponding the SHG image

In order to validate the sensitivity of SHG microscopy to structural irregularities, a spatial correlation analysis was performed against the established CL benchmark. Figure 6(a) presents the SHG intensity mapping of the GaN-on-Si heterostructure, where pronounced dark regions correspond to areas exhibiting a depressed nonlinear optical response. Regions with depressed SHG intensity are found to spatially correspond to areas with increased structural irregularity, suggesting that local defect-related perturbations may affect the effective second-order nonlinear susceptibility $\:{\boldsymbol{\chi\:}}^{\left(2\right)}$ and reduce the coherent nonlinear polarization within the focal volume, thereby modulating the measured SHG intensity. According to studies by Sun et al. and Angerer et al., high-density crystalline defects may induce local lattice disorder and degrade the long-range crystalline order. Such local perturbations may reduce the effective non-centrosymmetric interaction volume and thereby contribute to the localized SHG suppression observed in the present work [18, 25]. To further examine whether these dark SHG regions are associated with defect-rich zones, we compared the SHG intensity map with the overlay of the CL-derived RDE/NBE ratio, as shown in Fig. 6(b). Areas of low SHG intensity consistently coincide with regions of high R_DE/NBE_, suggesting an increase in deep-level nonradiative recombination associated with point defects and dislocation-related defect centers. This spatial correspondence suggests that the depressed SHG contrast is sensitive to defect-related regions; however, it should not be taken as direct evidence of a single physical mechanism. In addition to defect-associated crystalline disorder, other local factors, such as strain variation [15], modulation of the effective nonlinear optical response within the excitation volume [17], and piezoelectric-field redistribution [18], may also contribute to the observed SHG contrast. On the other hand, we evaluated the spatial agreement between the SHG anomaly map and the CL-derived RDE/NBE defect reference using several geometric metrics. The area ratio of 1.28 indicates that the SHG result slightly overestimates the defect-affected regions. This difference may be partly attributed to the different probing depths and contrast mechanisms of SHG and CL. Since the TDs extend vertically across the epitaxial layers, a partial displacement between SHG suppression and CL emission contrast is expected. The pixel-wise accuracy between the SHG anomaly map and the CL defect reference reaches 0.775, indicating good cross-modality spatial agreement. Overall, these results suggest that SHG suppression can serve as a reliable defect-sensitive optical signature for identifying optically active defect-related regions in GaN epilayers, although the present results do not allow a complete separation of the individual physical factors contributing to the SHG modulation.

### Comparative analysis with PLAD and object-centric detection models for SHG image

**Fig. 7:**
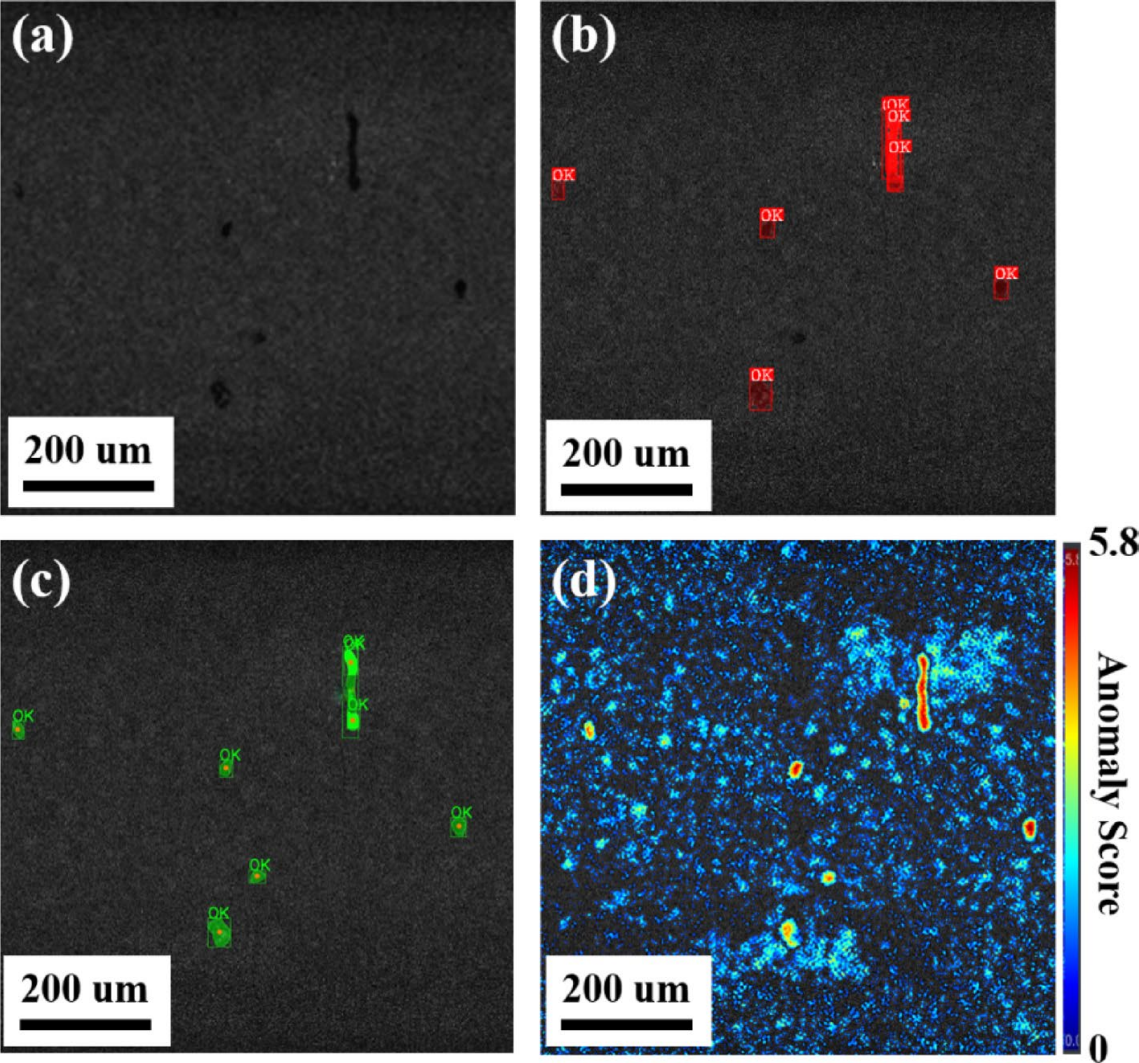
Comparison of defect detection methods on SHG images of the GaN on Si epilayer: **a** SHG image; detection results using **b** YOLOv8; **c** Mask R-CNN; **d** PLAD

Figure 7(a) shows the SHG image of the GaN-on-Si epilayer, serving as the structural contrast baseline. Figure 7(b)-(d) present the defect detection results of three detection approaches (YOLOv8, Mask R-CNN, and PLAD, respectively) applied to the same SHG image input. The object-based detectors, YOLOv8 and Mask R-CNN, successfully identify prominent defect zones with sharp SHG intensity drop-offs, leveraging architectures optimized for high-contrast, well-bounded features [26–28]. However, in regions where SHG contrast is gradual or the signal degradation is weak and spatially diffuse, these detectors may have limited ability to generate bounding boxes or segmentation masks because the edges are ambiguous and the anomalies do not conform to well-defined object shapes. In comparison, PLAD produces a dense anomaly score map that continuously highlights both high-contrast and low-contrast defect regions. To visualize the spatial distribution of the anomaly intensity, a pseudo-color map was generated based on the computed anomaly values. The anomaly scores were normalized between the global minimum and maximum values across the dataset prior to color mapping to ensure consistent visualization across samples. A threshold of 1 was applied to selectively highlight regions that exhibit significant deviations. Pixels with anomaly values below 1 were suppressed and displayed as background (black), while those exceeding the threshold were assigned colors using a Jet colormap ranging from blue (low) to red (high). Since PLAD performs pixel-wise normal modeling and deviation scoring, it can detect subtle symmetry breaking, phase perturbations, or micro-strain variations that may not appear as pronounced intensity decreases. This difference in detection behavior suggests that PLAD can provide improved sensitivity for identifying low-contrast or spatially diffuse optical defects. Object-based detectors are primarily designed for well-defined object boundaries, which may make detection of diffuse contrast variations more challenging. In contrast, PLAD is not constrained by predefined bounding geometry and can therefore be applied to irregular, small, or low-contrast anomalies. It should be noted, however, that PLAD identifies statistical deviations from nominal SHG patterns and therefore may also respond to non-defect-related contrast sources, such as surface contamination, local thickness variation, surface non-uniformity, or measurement-induced intensity fluctuations. As a result, not all anomaly signals can be directly attributed to intrinsic structural defects. In the present work, these effects were reduced through image preprocessing, selection of relatively uniform normal regions, and spatial comparison with the CL derived defect reference map. Overall, the results shown in Figure. 7 indicate that combining SHG imaging with PLAD can provide improved spatial representation of nonlinear optical defect signatures compared with object-level models, particularly for semiconductor optical inspection tasks where subtle defectivity is important.

### Quantitative performance evaluation and spatial fidelity analysis

In order to compare the detection performance, precision, recall, F1-score, and intersection over union (IoU) were calculated using the CL-derived R_DE/NBE_ defect map as the reference. The results are summarized in Table 1. Among the three methods, PLAD shows the best overall performance. In particular, PLAD achieves a recall of 0.3486 which is much higher than YOLOv8 (0.0321) and Mask R-CNN (0.0433) indicating improved sensitivity to weak and spatially distributed defect regions. The precision value of PLAD is also higher, leading to a significantly improved F1-score. Furthermore, the IoU of 0.2832 confirms better spatial overlap with the reference defect map. These results suggest that pixel-level anomaly modeling is more suitable for capturing distributed SHG defect patterns. Although PLAD outperforms YOLOv8 and Mask R-CNN in terms of IoU and recall, the absolute detection performance is still limited in the present study. Such improvement may be achieved through threshold optimization, integration of multi-scale or multi-modal features, and possible ensemble-based strategies.

**Table 1:** Quantitative comparison of defect detection performance using the CL-derived R_DE/NBE_ defect map as reference

Method	Precision	Recall	F1-score	IoU
YOLOv8	0.3019	0.0321	0.0580	0.0299
Mask R-CNN	0.4187	0.0433	0.0786	0.0409
PLAD	0.6015	0.3486	0.4414	0.2832

**Fig. 8:**
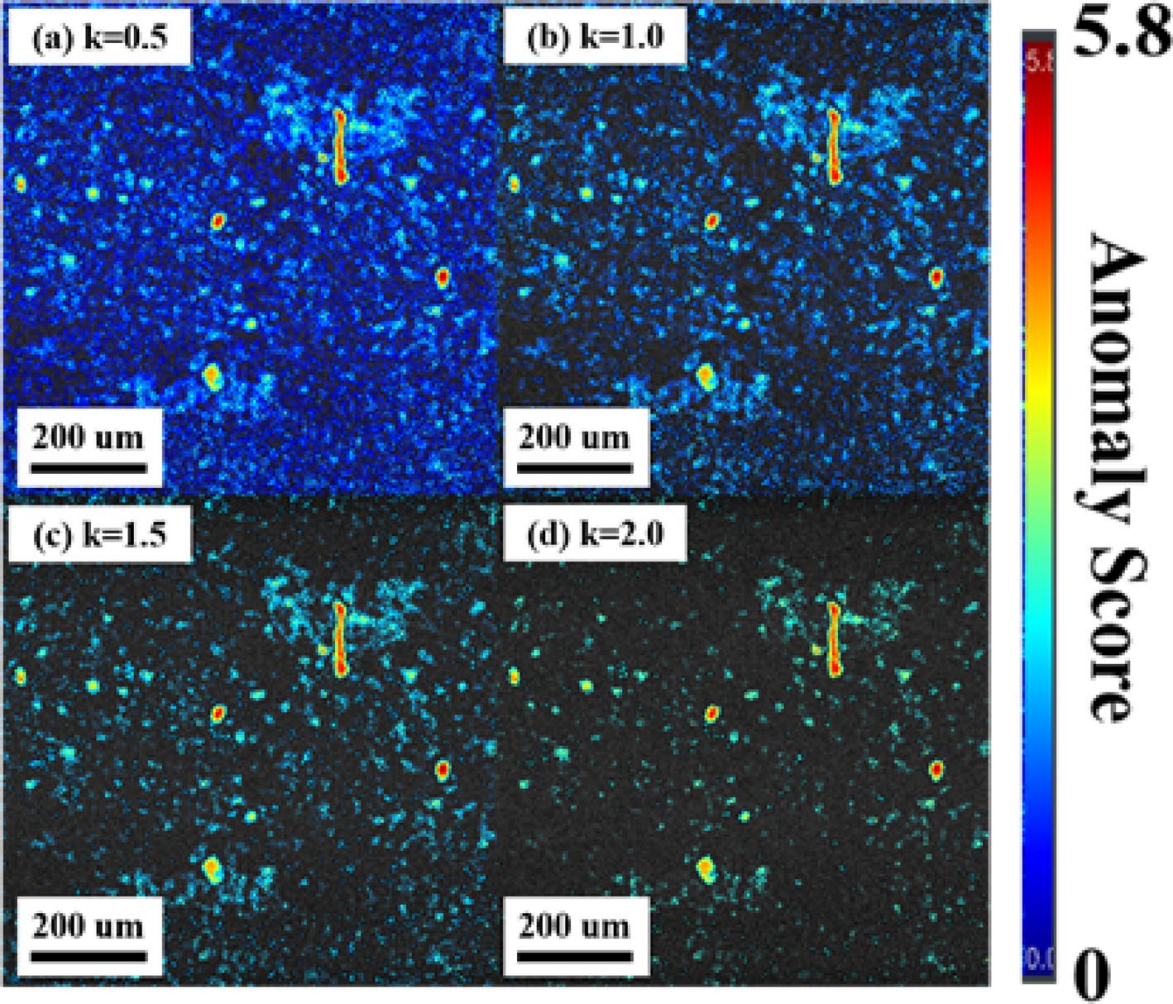
Threshold dependent segmented anomaly maps obtained from the PLAD analysis under different anomaly-score thresholds of **a** 0.5, **b** 1.0, **c** 1.5, and **d** 2.0.

In order to justify the selected anomaly-score threshold, a threshold sensitivity analysis was carried out using four threshold values (k = 0.5, 1.0, 1.5, and 2.0). The corresponding segmented anomaly maps are presented in Fig. 8. At k = 0.5, the segmented regions become noticeably enlarged, indicating that more anomaly pixels are retained, but also that more background responses are included, resulting in over-segmentation. In contrast, at higher thresholds of 1.5 and 2.0, only a limited number of strong anomaly regions remain, suggesting that weak but meaningful defect-related signals are suppressed. Among the tested conditions, k = 1.0 provides a more balanced segmentation result, preserving fine defect features without obvious expansion into the background.

This trend is quantitatively reflected in Table 2. At a threshold of 0.5, the true positive rate reaches 1.0, but the false positive rate also increases markedly to 0.5469, and the IoU remains relatively low at 0.1363. This indicates that the threshold is too low and results in substantial over-detection. In contrast, when the threshold is increased to 1.5 and 2.0, the false positive rate decreases to 0, whereas the true positive rate drops significantly to 0.1167 and 0.0441, respectively. The corresponding IoU values also decrease to 0.1167 and 0.0441. These results suggest that higher thresholds can effectively suppress background responses, but may also remove weak yet meaningful defect-related signals.

**Table 2:**
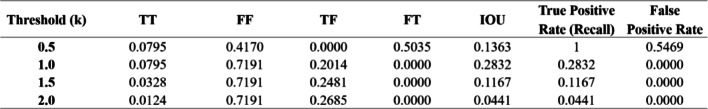
Quantitative comparison of segmentation performance under different anomaly score thresholds

**Fig. 9:**
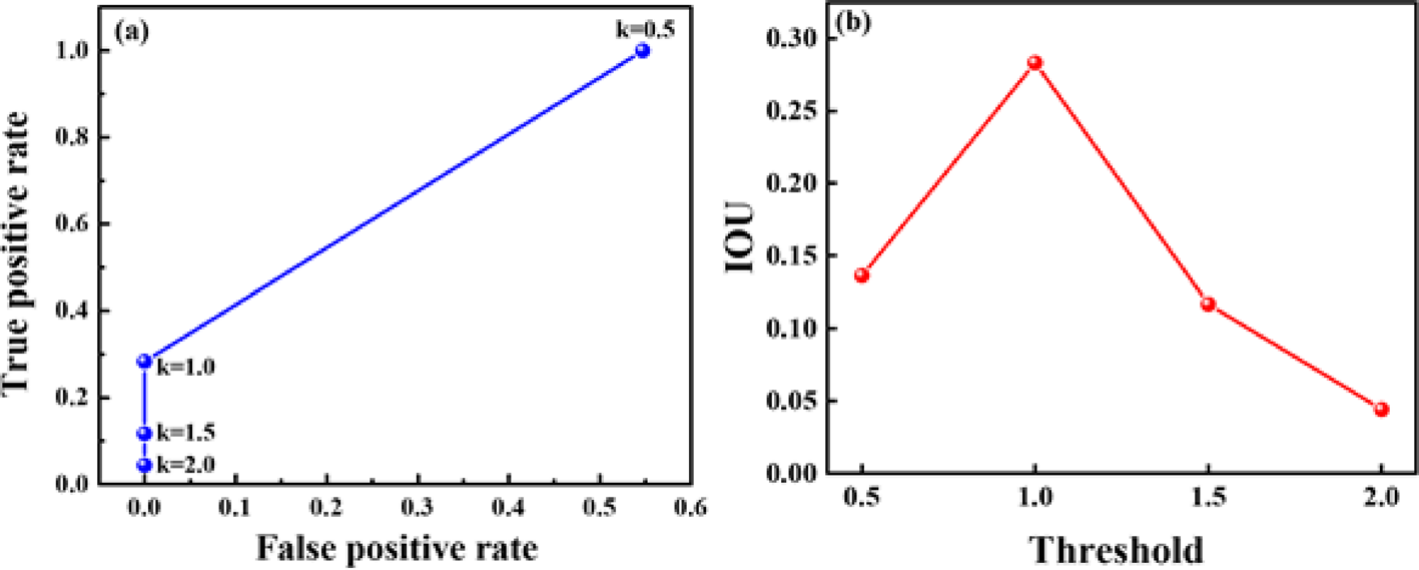
Threshold-dependent variation of **a** segmentation performance metrics and **b** IOU for PLAD based anomaly detection

Figure 9(a) shows the threshold-dependent TPR-FPR relationship of the PLAD results under different anomaly-score thresholds. It can be seen that a lower threshold increases the true positive rate, but also causes a higher false positive response. As shown in Figure 9(b), the IoU reaches the highest value of 0.2832 at threshold = 1.0, suggesting the best spatial agreement with the CL-derived defect reference among the tested conditions. Therefore, threshold = 1.0 appears to provide the most suitable balance between background suppression and retention of physically meaningful fine-scale anomaly regions. These additional analyses indicate that the threshold used in the main manuscript was supported by a systematic threshold-dependent evaluation.

### Implications for SHG-based defect inspection

**Fig. 10:**
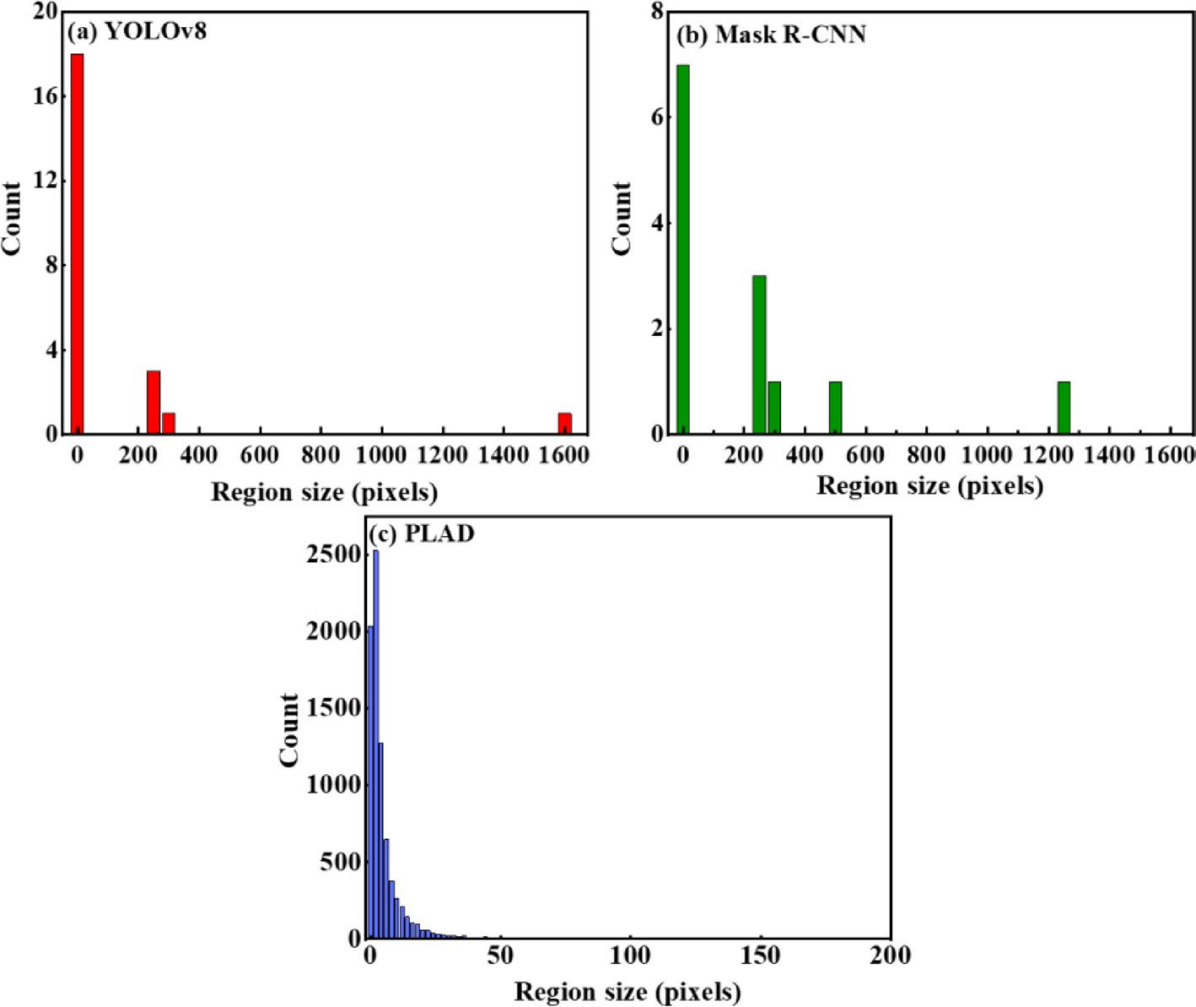
Histogram of defect region sizes obtained using 8-connected component analysis for detections generated by: **a** YOLOv8, **b** Mask R-CNN, and **c** PLAD

Figure 10 presents the defect region size histograms obtained via the 8-CCL technique, providing a quantitative assessment of detection sensitivity across varying length scales. The statistical distributions reveal that the supervised object detectors, YOLOv8 and Mask R-CNN, primarily identify a limited number of large-scale defect zones, with most detected regions exceeding 200 pixels. Although a negligible number of regions below the 50-pixel threshold appear in Figs. 10(a) and 10(b), these instances are attributed to mask fragmentation or boundary artifacts at the ambiguous edges of high-contrast defects rather than the systematic detection of micro-scale features. This behavior is inherent to their architectures, which prioritize high-contrast bounding box regression and mask proposals, inherently filtering out subtle or spatially diffuse signal variations as background noise. In comparison, the PLAD framework demonstrates sensitivity to fine-grained anomalies, successfully identifying over 5,000 distinct regions smaller than 50 pixels as shown in Fig. 10(c). This substantial population of micro-defects aligns with the subtle lattice irregularities previously verified by the R_DE/NBE_ mapping in the CL ground truth. By bypassing geometric constraints and utilizing pixel-wise modeling, PLAD effectively resolves vast populations of micro-scale optical perturbations that remain inaccessible to conventional object-level models. Consequently, this capability provides a significantly more comprehensive and high-resolution statistical representation of crystalline degradation within AlGaN/GaN heterostructures.

A summary of the comparative analyses given in Table 3, highlights the fundamental differences among the three detection paradigms. YOLOv8, as a bounding box-based detector, is optimized for computational speed and coarse object localization, but may have limited sensitivity to capture diffuse or weakly contrasted anomalies. Mask R-CNN provides improved segmentation fidelity by generating object-level masks; however, its reliance on region proposals still limits sensitivity to fine-grained defects. In contrast, the PLAD framework operates directly at the pixel level, enabling the detection of subtle, small-area anomalies (< 50 pixels) that are often overlooked by object-centric models. Consequently, the integration of PLAD with SHG imaging enables a more comprehensive micro-scale scalable defect mapping strategy, offering particular value in pre-fabrication quality assurance for GaN-on-Si heterostructures.

**Table 3:** Comparison of defect detection approaches applied to SHG imaging of GaN-on-Si heterostructures

Method	Output representation	Key strength	Key limitation	Typical detected region size	Use case
YOLOv8	Bounding boxes (object-level)	Fast inference; effective for high-contrast, well-bounded defect zones	Less sensitive to weak, diffuse contrast; coarse geometry	> 200 pixels	Rapid screening; coarse localization
Mask R-CNN	Instance masks (object-level)	Provides segmentation masks for prominent regions	Performance depends on clear edges and proposals; may miss faint regions	100–800 pixels	Segmentation of prominent defect zones
PLAD	Pixel-level anomaly score map	Sensitive to low-contrast and spatially distributed anomalies; fine-grained mapping without manual labels	Requires “normal” data modeling; threshold selection affects visualization	< 50 pixels (many regions)	Fine-grained mapping of subtle; diffuse anomalies

## Conclusion

In this study, we developed a non-destructive optical inspection framework that integrates SHG microscopy with PLAD to achieve high-resolution defect analysis in GaN-on-Si heterostructures. Our results show that the PLAD model provides improved sensitivity to low-contrast and micro-scale crystalline irregularities, with improved spatial representation compared with YOLOv8 and Mask R-CNN. Through cross-validation with CL spectral mapping, we observed that regions of suppressed SHG intensity are spatially consistent with areas of elevated defect-related luminescence (R_DE/NBE_), supporting the correlation between nonlinear optical signals and structural defect centers. Furthermore, statistical analysis using 8-CCL indicates that PLAD can identify thousands of micro-anomalies (< 50 pixels) that are difficult to detect using standard supervised models. This synergistic combination of nonlinear optical imaging and semi-supervised pixel-level learning enables efficient micro-scale defect mapping without the need for destructive etching or complex sample preparation. This work demonstrates a practical inspection approach that combines SHG microscopy with pixel-level anomaly modeling. By correlating SHG modulation with CL-derived defect density metrics, we present a non-destructive strategy for defect-related regions characterization in III-nitride heterostructures. The proposed SHG-PLAD framework provides a practical and non-destructive approach for crystalline defect characterization and structural quality assessment in AlGaN/GaN heterostructures.

## Data Availability

The datasets generated and/or analyzed during the current study are available from the corresponding author on reasonable request.
